# The Neuroprotective Effect of Lithium in cannabinoid Dependence is Mediated through Modulation of Cyclic AMP, ERK1/2 and GSK-3β Phosphorylation in Cerebellar Granular Neurons of Rat

**Published:** 2015

**Authors:** Hamid Reza Rahimi, Mohammad Hossein Ghahremani, Ahmad Reza Dehpour, Mohammad Sharifzadeh, Shahram Ejtemaei-Mehr, Ali Razmi, Seyed Nasser Ostad

**Affiliations:** a *Department of Toxicology and Pharmacology, Faculty of Pharmacy and Pharmaceutical Sciences Research Center, Tehran University of Medical Sciences, Tehran, Iran. *; b *PharmaceuticsResearch Center, Institute of Neuropharmacology, Kerman University of Medical Sciences, Kerman, Iran. *; c *Department of Toxicology and Pharmacology, Faculty of Pharmacy, Kerman University of Medical Sciences, Kerman, Iran.*; d *Experimental Medicine Research Center, Department of Pharmacology, School of Medicine, Tehran University of Medical Sciences,Tehran, Iran. *; e *Medicinal Plants Research Center, Institute of Medicinal Plants, ACECR, Karaj,Iran.*

**Keywords:** Dependence, WIN 55, 212-2, Cerebellar granular neurons, *In-vitro*, Cyclic AMP

## Abstract

Lithium (Li), a glycogen synthase kinase-3β (GSK-3β) inhibitor, has used to attenuate the cannabinoid-induced dependence/withdrawal signs, but molecular mechanisms related to this are unclear. Recent studies indicate the involvement of upstream extracellular signal kinase1/2 (ERK1/2) and downstream GSK-3β pathways in the development of cannabinoid-induced dependence. This is mediated through cannabinoid receptor 1 (CB1) enriched in cerebellar granular neurons (CGNs). Accordingly, the present study aimed to investigate the mechanism of modulatory/neuroprotective effects of Li on a cannabinoid agonist (WIN 55,212-2 (WIN))-induced dependence, through quantitative analysis of some involved proteins such as ERK1/2, GSK-3β and related signaling pathways including their phosphorylated forms; and cAMP level as the other molecular mechanisms leading to dependence, in CGNs model. The CGNs were prepared from 7-day-old Wistar rat pup in a 12-well plate, pretreated with Li (1mM) and an ERK1/2 inhibitor SL327 (SL, 10 µM). The WIN (1 µM) was added 30 minutes prior to treatment and AM251 (AM, 1 µM), as a cannabinoid antagonist was co-treated with WIN. The cAMP level, as an indicator of cannabinoid-induced dependence, was measured by ELISA following forskolin (FSK) stimulation. Western blot analyses determined the phosphorylated forms of ERK1/2 (p-ERK1/2), GSK-3β (p-GSK-3β) as well as their total expressions in various treatment times and doses in CGNs. WIN alone could down regulate the cAMP/p-ERK1/2 cascade compared to AM treatment. However, P-GSK-3β was up-regulated with Li and WIN or with SL and Li pretreatment to AM-induced cellular response, which was the highest 60 minutes after CGNs exposure. Results further suggested the potential role of Li pretreatment to diminish the development of cannabinoid-induced dependence/neuronal injury through possible mechanisms of modulating the cAMP/p-ERK1/2 cascade independent of p-GSK-3β signaling pathway* in-vitro*.

## Introduction

**Figure 1:**
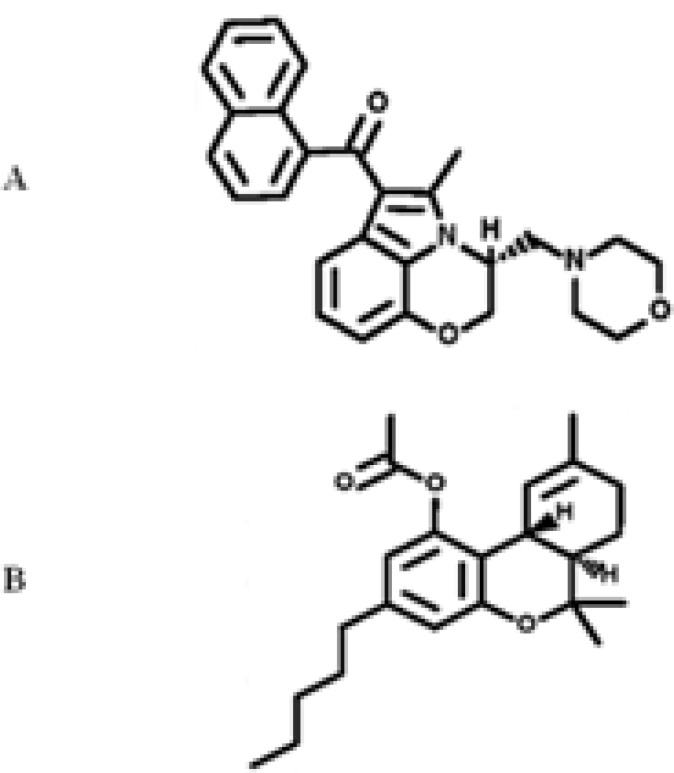
The structure of synthetic cannabinoid agonists WIN 55,212-2 (WIN). Molecular formula (MF) of C_27_H_26_N_2_O_3_ and molecular weight (MW) of 426.51 (A) and tetrahydrocannabinol (THC), MF of C_21_H_30_O_2_, and MW of 314.46 (B) drawing by PubChem program (http://www.ncbi.nlm.nih.gov/pccompound).

Cannabinoids include plant-derived phytocannabinoids principally found in *Cannabis sativa* (marijuana) (1), and endocannabinoids, which are produced naturally in body, have been implicated in various neurobiological processes including anxiety, movement control, and modulation of fear responses, cognition, pain relief, learning and memory (1-3). Furthermore, cannabinoids have been involved in neurodegenerative diseases including Alzheimer’s disease (AD) and Parkinson’s disease (PD); and they have promoted neuronal survival in cerebral ischemia or trauma (3-6). WIN 55,212-2 (WIN), the third category of cannabinoids, is a synthetic cannabimimetic aminoalkyl indole (AAI) derivative (Figure 1A) with similar effects to tetrahydrocannabinol (THC, Figure 1B), but with an entirely different structure (Figure 1). WIN is a full agonist of CB1 cannabinoid receptor and has a much higher affinity than THC for this receptor (7). It has been indicated that the development and expression of dependence and tolerance to most of the cannabinoid effects are due to its pharmacodynamic properties mediated through cannabinoid receptors (8).

Cannabinoid receptors belong to the G-protein-coupled receptors (GPCR) family, which consists of CB1 and CB2 and signal via Gαi/o proteins. CB1 is highly expressed in neuronal tissue such as hippocampal formation, basal ganglia and cerebellum (9). Furthermore, the cerebellum and to a lesser extent hippocampus and amygdale in the brain section are mostly participate in the behavior expression of cannabinoid dependence (10).Based on the development of cerebellar granule neurons (CGNs), the best time to culture these neurons from rats is postnatal 7-9-days-old rats, when the neurons are still developing, but have begun to express CB1 receptors and the cell number is adequate for primary culture (11, 12).Therefore, the rat CGNs provides an excellent model to study the cellular or molecular mechanisms of neuronal survival, toxicity, apoptosis and maintenance (13). However, CB2 is mainly located in peripheral tissues and is important in immune system (13, 14). It has been demonstrated that a CB1 receptor regulate a cluster of intracellular and/or molecular events including phospholipase C (PLC) activation as well as diacylglycerol (DAG) and inositol triphosphatase (I3P) activation, increasing intracellular calcium (Ca^2+^),opening of potassium (K^+^) channels, inhibition of adenylyl cyclase (AC) activity, and activation/in-activation of different protein kinases (PKs) such as extracellular signal-regulated kinases (ERK), PKC, glycogen synthase kinase-3β (GSK-3β), focal adhesion kinase (FAK), and phosphatidyl inositol-3-kinase (PI3K) (14-16). Cyclic adenosine mono phosphate (cAMP) cascade, which could regulate by AC and PKA enzymes, plays an important role among these signaling pathways in acute and chronic cannabinoid actions (17). Since its up-regulation, following cannabinoid antagonists such as AM251 (AM) has been correlated to the severity of expression of the withdrawal signs/neuronal excitation in cannabinoid-treated models (17-19).

Besides, activation of ERK1/2 and GSK-3β pathways has been implicated in the regulation of cannabinoid withdrawal signs and the development of cannabinoid-induced dependence. It has demonstrated that pretreatment of animals with SL327 (SL), a specific inhibitor of mitogen-activated protein kinase (MAPK)/ERK kinase (MEK) inhibitor, prevented the development of cannabinoid tolerance to induce hypolocomotion (20, 21). On the other hand, GSK-3β is downstream protein of ERK in which different transcription factors (TFs) can be regulated via its activation, including cAMP response element binding proteins (CREB), nuclear factor-kβ (NF-kβ), β-catenin and p53 (22, 23). Therefore, it may be regulated the expression of special genes involved in inducing of cannabinoid dependence. Furthermore, GSK-3β dysfunction is related to the pathophysiology of mood disorders, schizophrenia, diabetes, cancer, inflammation, AD; and may be involved in cannabinoid-induced dependence resulting to induce withdrawal signs, excitation and neuronal plasticity/injury status (24-26). GSK-3β can be pharmacologically inhibited by lithium (Li) as a mood stabilizer through direct binding to the ATP-dependent magnesium (Mg^2+^)-sensitive catalytic enzyme, or indirectly through enhanced phosphorylation of GSK-3β by activation of PKA, PI3K/Akt signaling pathways (PKB) and phosphorylation of PKC at Ser 9 (22, 23).

In this regard, Li (1mM) has been shown to have a preventive effect on the tolerance to cannabinoid in guinea pig ileum *in-vitro *(17). On the other hand tolerance and dependence are associated together and are like two sides of the same coin. Since, Li is effective to attenuate the cannabinoid-induced dependence/withdrawal signs. Despite recent studies illustrating the role of some proteins such as ERK1/2 and GSK-3β or related signaling pathways including their phosphorylation forms and cAMP level in cannabinoid-induced dependence, the potential modulatory/therapeutic effects of Li on this, to know related molecular mechanisms/signaling pathways with regard to cannabinoid antagonist as not been identified. Therefore, we have purposed to develop a model of cannabinoid agonist (WIN)-induced dependence in CGNs to elucidate some molecular mechanisms involved.

## Experimental

*Reagents and Antibodies*

WIN 55,212-2 (WIN) was purchased from Cayman Chemicals (Ann Arbor, MI, USA). SL327 (SL) and AM251 (AM) were purchased from Tocris Bioscience (Tocris Biosciences, UK). RPMI 1640 and DMEM media, FBS (Fetal Bovine Serum), trypsin, penicillin and streptomycin were purchased from Biosera (Austria). Western blot detection kit and nitrocellulose membrane were prepared from Amersham (GE Healthcare, UK). Antibodies against p-ERK1/2, ERK1/2 (also known as p44/42 MAPK), p-GSK-3β and GSK-3β were purchased from Cell Signaling Technology (Danvers, MA, USA). β-actin antibody was purchased from Santa Cruz Biotechnology (Santa Cruz, CA, USA). Direct Cyclic AMP Enzyme Immunoassay Kit from Assay Designs (Ann Arbor, MI, USA). Lithium chloride and all other reagents were from Merck (Germany).

*Animals*

Animals Wister albino rats, weighing 200-250 g, were obtained from a single breeding colony of Pasteur Institute, Tehran, Iran. Animal experiments were conducted in accordance with current ethical regulations on animal research in Tehran University of Medical Sciences, and the investigation conformed to the “Guide for the Care and Use of Laboratory Animals” published by the United States National Institutes of Health (NIH Publication, 8th Edition, 2011). Animals (including one male and two females in each cage) were randomized and housed under standard laboratory conditions (temperature of 20 ± 2°C, relative humidity of 40-45% and light-dark cycle of 12:12 h) and free access to standard laboratory food and water.

*Cell culture*

**Figure 2:**
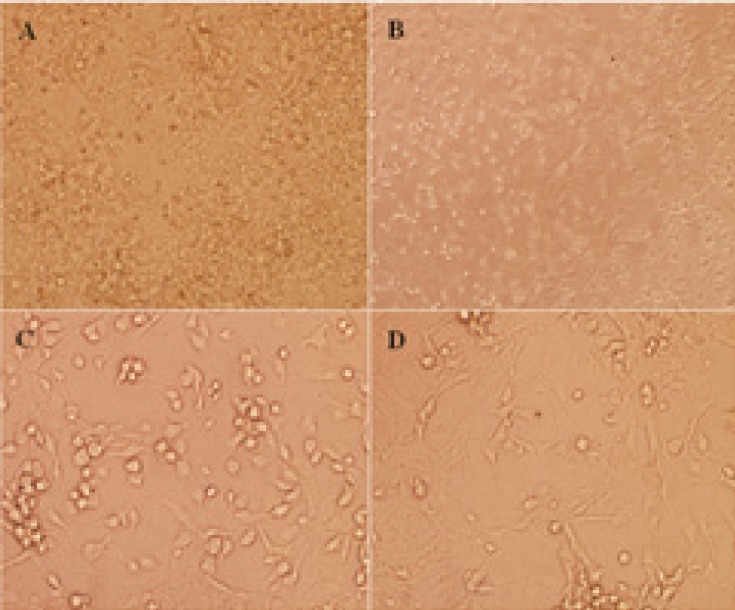
The dissociated cerebellar granular cells (CGNs) from cerebellum of postnatal 7-day-old Wistar rat pups. CGNs after 24 h seeded in 12-well culture plate were presented with × 100 magnification (A). CGNs after 24 h following cytosine arabinoside (Ara-C) added with × 100 (B) and × 400 magnification (C), respectively, using light microscope were indicated. CGNs at day 5 which all pharmacological interventions were started were presented with × 400 magnification (D).

Postnatal7-day-old Wistar rat pups were firstly sprayed with 95% ethanol and sacrificed by decapitation, and then cut off heads into Petri dishes on ice containing cold phosphate buffered saline (PBS). Meninges were removed from each isolated cerebellum by gently teasing with two forceps and placed into clean dish with cold PBS on ice. Cerebella were collected, rinsed in HBSS-BSA, minced, trypsinized and triturated to dissociated CGNs as described (11, 12). Briefly, DMEM containing 10% fetal bovine serum (FBS), was added to stop the digestion. CGNs were then seeded on poly-D-lysine (10 µg/ml) coated polystyrene 12-well tissue culture plates at 3.2×10^5^/cm^2^ density in DMEM high glucose medium containing 10% FBS, 25 mM KCl, penicillin (100 IU/ml), and streptomycin (100 mg/ml) at 37ºC in a 5% CO_2_ humid atmosphere. After 24 h, 10 µM of cytosine arabinoside (Ara-C) was added to inhibit the growth of non-neuronal cells. The medium was replaced after 24 h with fresh DMEM medium and lower FBS (5%) was replaced. All pharmacological interventions were started at day 5 when CGNs had developed their neurite (Figure 2). The medium was then changed to fresh DMEM medium without FBS, for 24 h prior to treatments, to lower the possible effect of serum on proteins expression.

*Treatment procedure*

CGNs were treated with WIN (1 and 2 µM) for 15, 30, 60 and 180 min. To study the effect of Li or SL and WIN, cells were pretreated with Li (0.3 and 1 mM) or SL (10 µM), 30 min before WIN exposure. AM (1 µM) was also co-treated with WIN to block its molecular or cellular response such as cAMP levels as cannabinoid-induced dependence/withdrawal indicator. The CGNs lysate was prepared 15, 30, 60 and 180 min after WIN treatment. In control (CTRL) group, distilled water (DW) as vehicle for Li or dimethyl sulfoxide (DMSO) for other drugs were used in equal volume to added drugs.

*Western blotting*

After incubation periods, CGNs were washed in PBS and lysed with lysis buffer containing 62.5 mM Tris HCl (pH 6.8), dithiothreitol 50 mM, glycerol 10% and bromophenol Blue 0.25% (W/V). The lysate were then collected in a microfuge tube and kept in -80ºC until use. After that, the lysates were boiled in water for 8 min and then the equal amount (40 µl) of protein samples were loaded into 12% SDS-PAGE. Separated proteins were transferred onto nitrocellulose membranes at 120 V for 60 min and blocked with 1% blocking solution, and then incubated with anti-p-ERK1/2, ERK1/2, p-GSK-3β and GSK-3β at 1:1000 dilution at 4°C, overnight. Blots were probed with anti β-actin antibody (1:2000) as internal CTRL. The blots were then washed three times, and probed with horseradish peroxidase (HRP)-conjugated goat anti-rabbit secondary antibody (1:10000) for 60 min at room temperature. Protein bands were detected on X-ray film by using a chemiluminescence kit, and then were quantified by densitometry using ImageJ software (USA) and normalized to β-actin band intensity. All data were presenting as mean ± SD of three independent experiments.

*Cyclic AMP assay*

CGNs were cultured at 3.2×10^5^/cm^2^ density in 12-well plate and treated with described method for 60 min (11, 27). Before, cAMP measurement, forskolin (FSK, 1 µM) was added for 10 min following the addition of WIN or Li into the CGNs medium, to induce the cAMP level. Then CGNs were washed, lysed in 0.1 M HCl (500 µl/well), and centrifuged at 600 g. Supernatants were collected for the cAMP assayed by using Direct Cyclic AMP Enzyme Immunoassay Kit, following to the manufacturer's instructions. Optical density (OD) was read at 405 nm immediately after addition of stop solution in room temperature. The intensity of the yellow color generated was inversely proportional to the concentration of cAMP in either standards or samples.

*Data analysis*

All data were presented as mean ± SD (standard deviation). The significance among the groups was calculated by one-way analyses of variance (ANOVA) followed by a Tukey post hoc test for multiple comparisons. Differences were considered statistically significant when p-value less than 0.05. 

## Results

*Effect of WIN treatment on ERK1/2 and GSK-3β pathways in CGNs*

**Figure 3:**
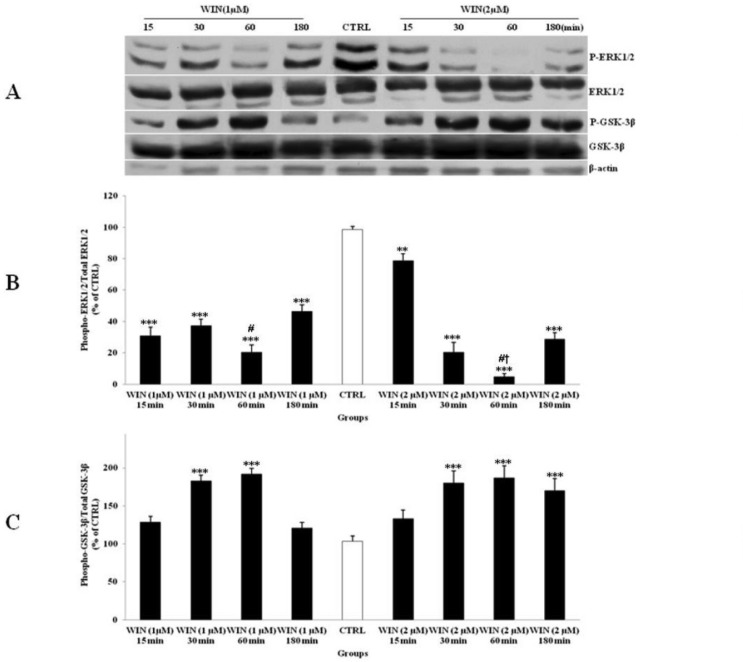
The effect of WIN 55,212-2 (WIN) as a potent cannabinoid agonist, on the phosphorylated-ERK1/2 (p-ERK1/2), total ERK1/2, phosphorylated-GSK-3β (p-GSK-3β) and total GSK-3β expression in cerebellar granular cells (CGNs). CGNs were treated with 1 and 2 µM of WIN and the cell lysates were prepared after 15, 30, 60 and 180 minutes. The protein expression was analyzed by western blotting. The p-ERK1/2 and ERK1/2 expression (A, B), and the p-GSK-3β and GSK-3β expression (A, C), were evaluated. The protein expression was normalized to β-actin as internal control (CTRL). The band intensity was measured and presented as the percent of un-treated cells (CTRL). All data were presented as Mean ± SD. Statistically significant values are presented as follows: *** p < 0.001, ** p < 0.01 and * p < 0.05, compared to CTRL; **#** p < 0.05, compared to respective time in two doses; **†** p < 0.001, compared the dose of 1 µM with the dose of 2 µM at 60 min exposure

The level of phosphorylated ERK1/2 (p-ERK1/2) was lower when cells were treated with WIN (Fig. 3A). On the other hand, WIN treatment was significantly increased phosphorylated-GSK-3β (p-GSK-3β) compared to CTRL (Figure 3B). The effects of WIN on p-ERK1/2 and p-GSK-3β started at 15 min and reached to its maximum after 60 min. This effect was increased when CGNs were treated with higher WIN concentration (2 µM). However, no significant changes were indicated between two described doses to up regulate the p-GSK-3β expression in 60 min of exposure. The p-ERK1/2 and p-GSK-3β returned to CTRL level after 180 min. These data indicated a time- and dose-dependent response of p-ERK1/2 expression by WIN in CGNs (Figure 3). WIN treatment had no significant effect on the total expression of ERK1/2 and GSK-3β. Based on these results, the effective dose of 1 µM and the time of 60 min of exposure were selected for the rest of the study.

*Effect of lithium treatment on ERK1/2 and GSK-3β pathways in CGNs*

**Figure 4:**
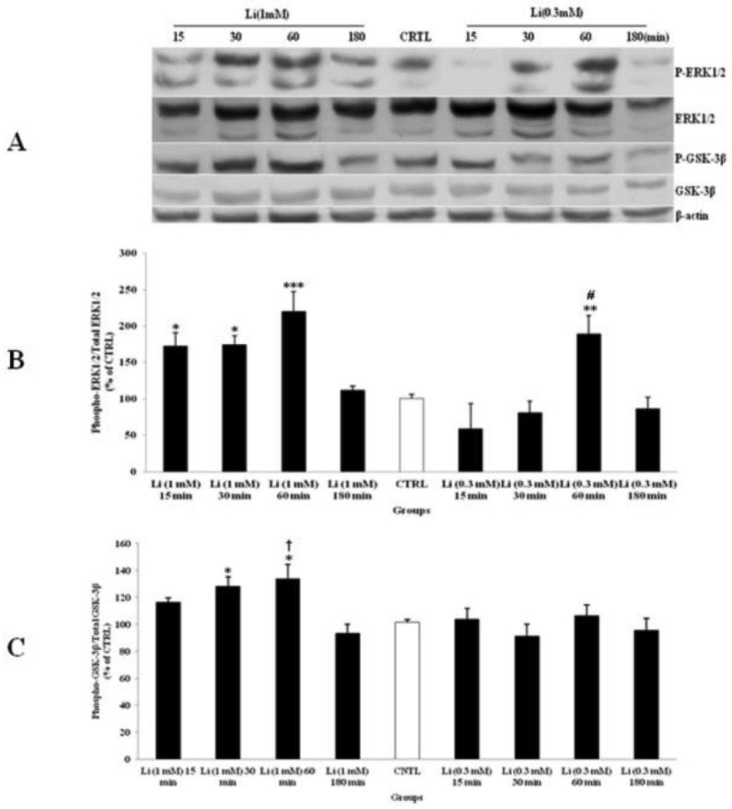
The effect of lithium (Li) as a mood stabilizer drug, on the phosphorylated-ERK1/2 (p-ERK1/2), total ERK1/2, phosphorylated-GSK-3β (p-GSK-3β) and total GSK-3β expression in cerebellar granular cells (CGNs). CGNs were treated with 0.3 and 1 mM of Li and the cell lysates were prepared after 15, 30, 60 and 180 minutes. The protein expression was analyzed by western blotting. The p-ERK1/2 and ERK1/2 expression (A and B), and the p-GSK-3β and GSK-3β expression (A and C), were evaluated. The protein expression was normalized to β-actin as internal control (CTRL). The band intensity was measured and presented as the percent of un-treated cells (CTRL). All data were presented as Mean ± SD. Statistically significant values are presented as follows: *** p < 0.001, ** p < 0.01 and * p < 0.05, compared to CTRL; **#** p < 0.05, compared to respective time in two doses; **†** p < 0.001, compared the dose of 0.3 mM with the dose of 1mM at 60 min exposure

The effect of Li treatment on CGNs was examined with two doses of 0.3 and 1 mM for 15, 30, 60 and 180 min. The CTRL group received equal volume of DW. Li increased the expression of p-ERK1/2 (Figure 4A and B) and p-GSK-3β (Figure 4A and C) significantly. The highest level of p-ERK1/2 and p-GSK-3β were occurred after 60 min of Li treatment. The phospho-protein level returned to CTRL level after 180 min. Furthermore, the dose of 1 mM has shown more induction of the expression of p-ERK1/2 and p-GSK-3β in comparison to the dose of 0.3 mM (Figure 4). Li had no significant effect on the total expression of ERK1/2 and GSK-3β in comparison to CTRL. Therefore, the effective dose of 1 mM of Li was selected for the rest of the study.

*Effect of SL327 and AM251 on ERK1/2 and GSK-3β pathways in CGNs*

**Figure 5:**
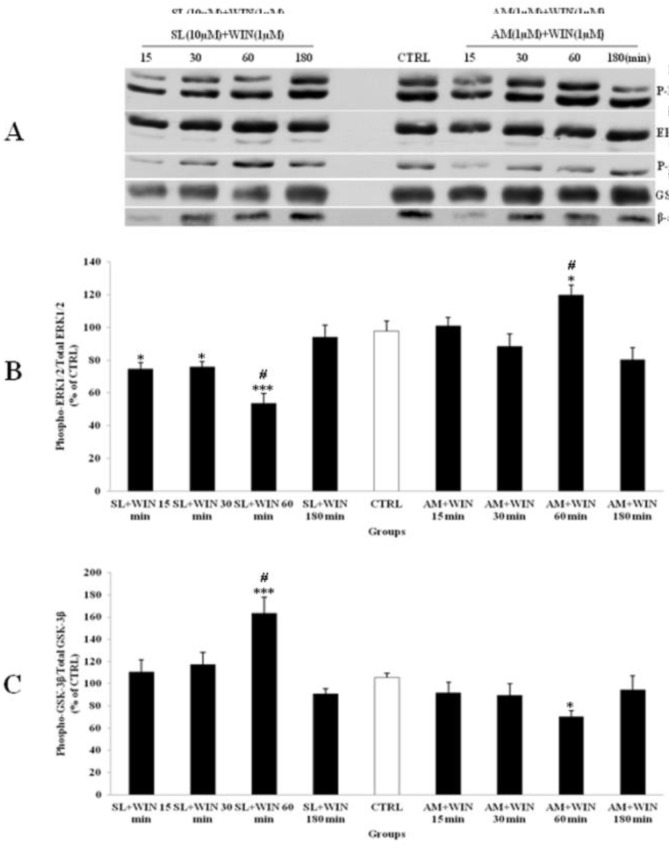
The effect of SL327 (SL) as a specific inhibitor of ERK1/2 pathway and AM251 (AM, 1 µM) as a cannabinoid antagonist on the phosphorylated-ERK1/2 (p-ERK1/2), total ERK1/2, phosphorylated-GSK-3β (p-GSK-3β) and total GSK-3β expression in cerebellar granular cells (CGNs). CGNs were pre-treated with SL (10 µM), 30 min before added WIN 55,212-2 (WIN, 1 µM) and AM was co-treated with WIN in CGNs 12-well plate and the cell lysates were prepared after 15, 30, 60 and 180 minutes. The protein expression was analyzed by western blotting. The p-ERK1/2 and ERK1/2 expression (A and B), and the p-GSK-3β and GSK-3β expression (A and C), were evaluated. The protein expression was normalized to β-actin as internal control (CTRL). The band intensity was measured and presented as the percent of un-treated cells (CTRL). All data were presented as Mean ± SD. Statistically significant values are presented as follows: *** p < 0.001, ** p < 0.01 and * p < 0.05, compared to CTRL; **#** p < 0.05, compared the 30 to 60 min exposure

SL pretreatment had small effect on p-ERK1/2 expression since WIN could decrease ERK1/2 phosphorylation within 60 min (Figure 5). On the other hand AM blocked the WIN-induced inhibition of p-ERK1/2 in CGNs (Figure 5A). Similarly, the p-GSK-3β expression was stimulated by WIN and SL pretreatment. However, the WIN induced p-GSK-3β increase was blocked by AM co-treatment (Figure 5B). As shown in the Figure 5 the change in the levels of p-ERK1/2 and p-GSK-3β also returned to CTRL after 180 min. However, no significant change was shown between the total level of ERK1/2 and GSK-3β expression. Thus, cannabinoid receptor is involved in WIN induced changes in phosphorylation of ERK1/2 and GSK-3β since both pathways was blocked by CB1 receptor antagonist.

*Lithium and SL327 pretreatment modified the ERK1/2 pathway independent to GSK-3β expression in CGNs*

**Figure 6:**
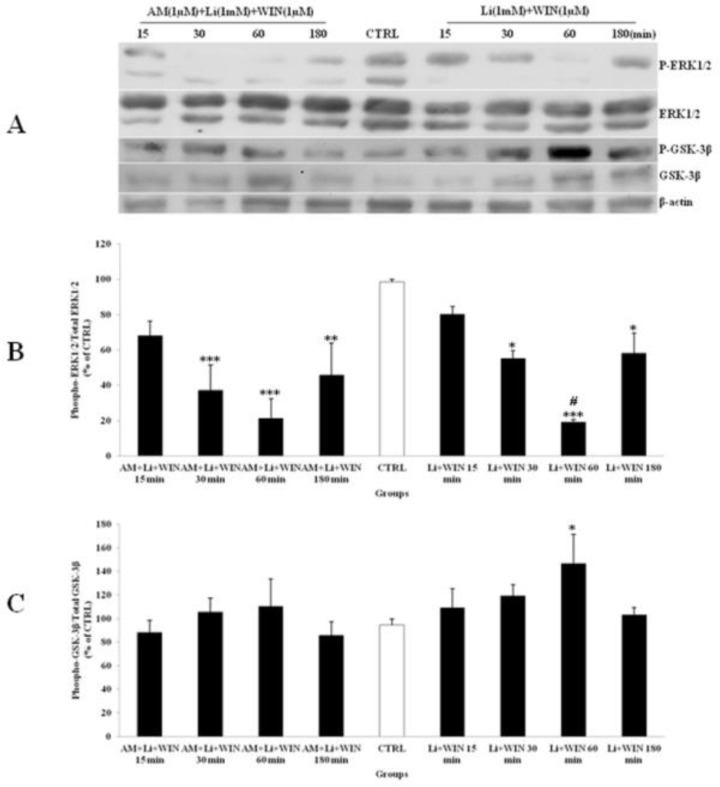
The effect of lithium (Li) pre-treated to WIN 55,212-2 (WIN) and AM251 (AM) on the phosphorylated-ERK1/2 (p-ERK1/2), total ERK1/2, phosphorylated-GSK-3β (p-GSK-3β) and total GSK-3β expression in cerebellar granular cells (CGNs). CGNs were pre-treated with Li (1mM), 30 min before added WIN (1 µM) and AM (1 µM), and the cell lysates were prepared after 15, 30, 60 and 180 minutes. The protein expression was analyzed by western blotting. The p-ERK1/2 and ERK1/2 expression (A and B), and the p-GSK-3β and GSK-3β expression (A and C), were evaluated. The protein expression was normalized to β-actin as internal control (CTRL). The band intensity was measured and presented as the percent of un-treated cells (CTRL). All data were presented as Mean ± SD. Statistically significant values are presented as follows: *** p < 0.001, ** p < 0.01 and * p < 0.05, compared to CTRL; **#** p < 0.05, compared the 30 to 60 min exposure

**Figure 7:**
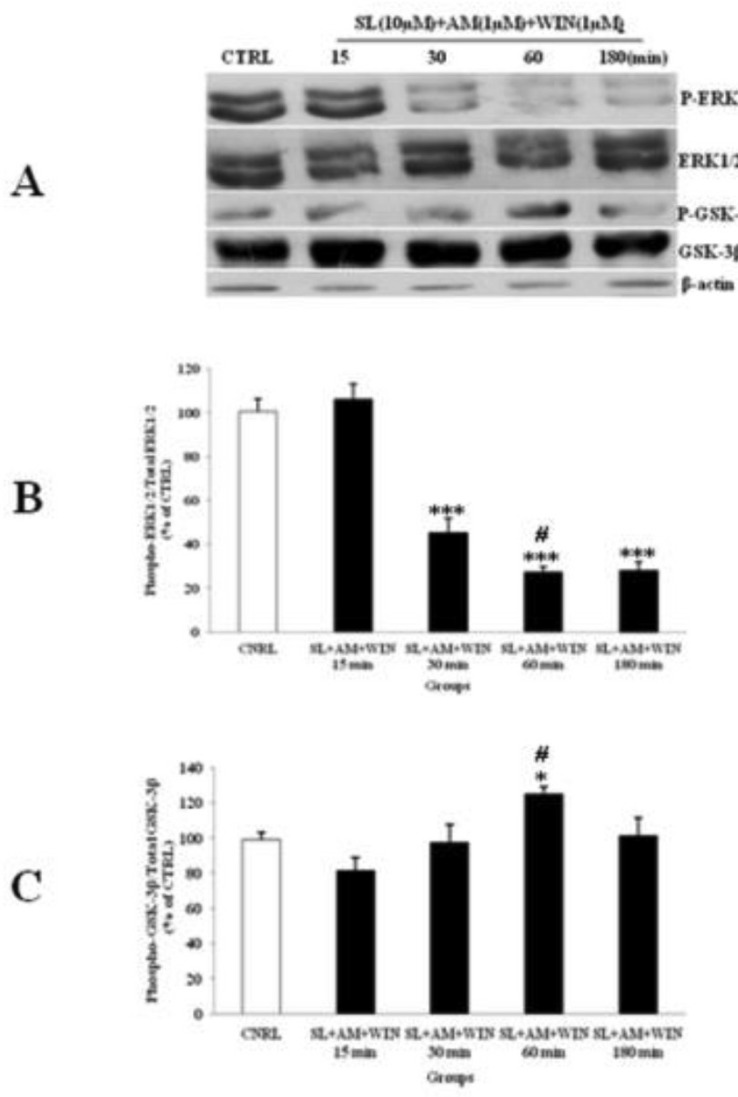
The effect of SL327 (SL) pre-treated to WIN 55, 212-2 (WIN) and AM251 (AM) on the phosphorylated-ERK1/2 (p-ERK1/2), total ERK1/2, phosphorylated-GSK-3β (p-GSK-3β) and total GSK-3β expression in cerebellar granular cells (CGNs). CGNs were pre-treated with SL (10 µM), 30 min before added WIN (1 µM) and AM (1 µM), and the cell lysates were prepared after 15, 30, 60 and 180 minutes. The protein expression was analyzed by western blotting. The p-ERK1/2 and ERK1/2 expression (A and B), and the p-GSK-3β and GSK-3β expression (A and C), were evaluated. The protein expression was normalized to β-actin as internal control (CTRL). The band intensity was measured and presented as the percent of un-treated cells (CTRL). All data were presented as Mean ± SD. Statistically significant values are presented as follows: *** p < 0.001, ** p < 0.01 and * p < 0.05, compared to CTRL; **#** p < 0.05, compared the 30 to 60 min exposure

Li (1mM) alone had up-regulated phosphorylation of ERK1/2 (Figure 4) and WIN (1µM) had decreased p-ERK1/2 (Figure 3). We tested the p-ERK1/2 expression in the presence of Li, SL and WIN to study their interaction. Our results indicated that Li partly blocked WIN effect on p-ERK1/2 level (Figure 6A), suggesting interaction of Li and cannabinoid receptor in ERK pathway. The AM treatment further lowered p-ERK1/2 in the presence of WIN and Li (Figure 6). Furthermore, the p-GSK-3β expression was increased with Li or WIN treatment alone (Figure 3, 4). CGNs treated with Li and WIN had higher p-GSK-3β expression suggesting an additive effect in these conditions (Figure 6B). AM treatment with Li and WIN combination exhibited increase in p-GSK-3β expression, but the level is lower than Li + WIN. This effect could be due to block of cannabinoid receptor with AM as antagonist (Figure 6). These data indicated that Li pretreatment could potentially modify the expression of p-ERK1/2 (Figure 6A and B) and p-GSK-3β (Figure 6A and C) level. Li and SL pretreatment can also inhibit the p-ERK1/2 expression. SL pretreatment can attenuate the up-regulation of p-ERK1/2 induced with AM exposure. Furthermore, p-GSK-3β level was increased by SL pretreatment, which was down-regulated with AM (Figure 7). However, they did not down-regulate the expression of p-GSK-3β level. Therefore, Li could modify the effect of cannabinoid through p-ERK1/2 cascade independent of p-GSK-3β pathways.

*Lithium and SL327 pretreatment modulated the cAMP level against AM251 cellular response*

**Figure 8:**
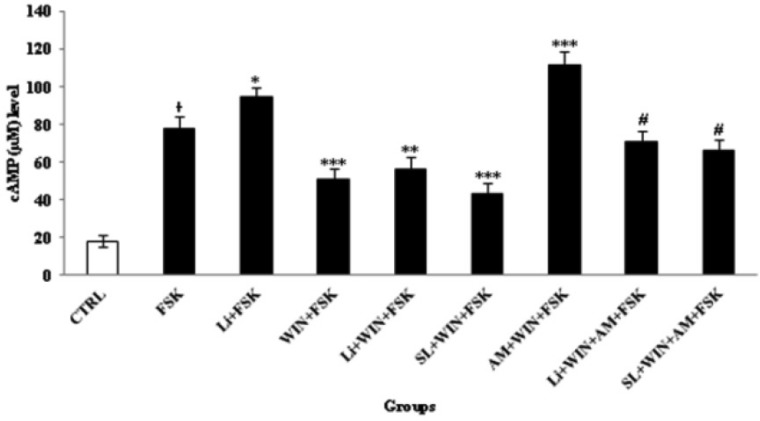
Cyclic AMP (cAMP) changes in study-treated groups in cerebellar granular neurons (CGNs). CGNs were cultured at 3.2 × 10^5^/cm^2^ density in 12-well plate and treated with described method for 60 min. Before, the cAMP measurement, forskolin (FSK, 1 µM) was added for 10 min following the addition of WIN 55,212-2 (WIN, 1 µM) or Lithium (Li, 1 mM) into the CGNs medium, for induction of cAMP level. The control (CTRL) group was CGNs without treatment. All data were presented as Mean ± SD. Statistically significant values are presented as follows: * p < 0.05, compared to CTRL, *** p < 0.001, ** p < 0.01 and * p < 0.05, compared to FSK group; **#** p<0.05, compared to AM (AM251, 1 µM) + WIN 55, 212-2 (WIN, 1 µM) + FSK (1 µM) group

A cAMP level was measured after 60 min as the indicator of cannabinoid-induced dependence/withdrawal in following of 10 min added of FSK (1µM) in treated groups (Figure 8). FSK increases the cAMP concentration as detected by cAMP ELISA Assay kit. Since the up-regulation of cAMP level was according to the intensity of cannabinoid abstinence. As shown in Fig 8 the cAMP level was inhibited with treatment of WIN. However, the cAMP level was up-regulated in co-treatment of WIN with AM. Furthermore, SL and Li pretreatment can modulate the cAMP level. Therefore, p-ERK1/2 and cAMP cascade were important in the development of cannabinoid-induced dependence. These data was in agreement with the neuroprotective role of Li to down or up-regulation of cAMP level, to maintenance the cannabis or bipolar disorder. P-ERK1/2, p-GSK-3β and cAMP cascade were interacting with each other to induce cannabinoid dependence. Therefore, Li can attenuate the development of cannabinoid-induced dependence/withdrawal underlying cAMP and p-ERK1/2 cascade independent of p-GSK-3β pathway.

## Discussion

Cannabinoids and opioids have displayed many similar responses, such as antinociception, hypolocomotion, hypothermia, as well as cross development of tolerance and physical dependence/withdrawal signs (28, 29). Furthermore, cannabis is the most widely used banned drug in the world, and only a few of cannabis users seek medical help with their addiction disorders (30). Suitable preclinical and clinical trial studies should be designed to treat cannabis disorders. Therefore, understanding the pure signaling pathways involved in the regulation and/or induction of cannabinoid dependence in an isolated especial neuronal cells model, such as CGNs in a preclinical study, will provide potent therapeutic drugs targeted to overcome this problem. Various signaling pathways, such as p-ERK1/2 and p-GSK-3β expression underlying cAMP cascade and have been reported to regulate the cannabinoid receptor and may also be involved in the development of cannabinoid to dependence (31-33). Recent studies have suggested Li for the treatment of cannabinoid-induced dependence or cannabis-use disorders (34, 35); however, its effective role and/or possible related molecular mechanisms have less clarity.

In the present study, we have demonstrated a time- and dose-dependent inhibition of the phosphorylation of ERK1/2 and induction of phosphorylation of GSK-3β with WIN as cannabinoid agonist. However, pretreatment of CGNs to WIN, with SL as a specific ERK pathway inhibitor and Li as a putative GSK-3β inhibitor, attenuate the cellular response to AM as a cannabinoid antagonist, such as cAMP elevation. In agreement with this study, cannabinoids can down-regulate the p-ERK1/2 pathway (21) and up-regulate the p-GSK-3β level *in-vitro *(10), in which the first pathway was in accordance with their anti-proliferative action and the second one can be tied to neuronal survival. With regard to the effect of Li on GSK-3β and ERK1/2 signaling pathways, it is reasonable to consider Li can be effective in modulating the cannabinoid-induced dependence through regulations of cAMP/p-ERK1/2 and p-GSK-3β pathways in the CGNs.

Since, studies have reported that Akt/PI3K signaling pathways regulated the p-GSK-3β up-regulation (10, 32) and cannabinoids can induce neuronal proliferation by CB1 receptor via PI3K/Akt activation and phosphorylation of GSK-3β (10). However, the regulation of the GSK-3β pathway with other kinase, such as ERK1/2 pathway, in cannabinoid-induced dependence is unclear. To evaluate the crosstalk of p-ERK1/2 and p-GSK-3β with regard to cannabinoid receptor activation, we inhibited ERK1/2 pathway using SL327 (MEK inhibitor). Interestingly, blocking the ERK activation resulted in inhibition of a WIN decrease in p-ERK expression and blocking of p-GSK-3β expression. These results suggested that ERK is up stream to WIN induced GSK-3β phosphorylation. On the other hand, ERK1/2 signaling can be down-regulated with cannabinoids (21). It has been reported that Ras/ERK signaling is essential for triggering the alteration in the CB1 receptor function, which may be responsible for the development of cannabinoid tolerance or dependence (20). Pretreatment of animals with SL327 fully prevented the development of tolerance to cannabinoid-induced hypolocomotion (20). It was reported that the presynaptic inhibitory action of CB1 receptors at corticostriatal synapses modulated the inhibitory effect of WIN on the activation of the ERK1/2 pathway by AM (36).

The CB1 agonist reduced cAMP production induced by isoproterenol (Isuprel) or FSK, and suppressed the phosphorylation of MAPK, such as P38 and ERK *in-vitro *(37). Moreover, cAMP up-regulation is mediated with basal and calcium-calmodulin-stimulated AC, since micro infusing the cAMP blocker Rp-8Br-cAMPS in the cerebellum of animal can markedly reduce both PKA activation and the somatic expression of cannabinoid withdrawal (18). Our results indicate that activation of the cannabinoid receptor inhibits FSK induced cAMP and this effect is blocked by AM. However, inhibition of ERK pathways by SL327 had no significant effect on cAMP inhibition by WIN, suggesting that cAMP is upstream to ERK activation.

The cAMP can crosstalk with ERK1/2 through GTPase Rap1 activation, which can activate or inhibit ERK1/2 signaling pathway in a cell-specific manner (33). Therefore, ERK1/2 in-activation can be regulated by cAMP/PKA cascade (18). In this regard, Li has little effect on WIN-induced inhibition of cAMP production. Since in GSK-3β is downstream to the ERK pathway following WIN activation, it is predictable that a Li block of p-GSK-3β has little effect on cAMP production.

Studies have reported the potential of Li in preventing the cannabinoid-induced dependence (8, 34, 35). GSK-3β is a putative target of Li, to which its inhibition and its downstream proteins, including reduction of both tau protein phosphorylation and amyloid-β42 production, was associated with the neuroprotective effect of Li reducing the risk of neurodegenerative diseases such as AD in subjects with bipolar disorder (38). However, its activation has a central role in drug-induced withdrawal (39). We have shown that Li alone, at doses of 0.3 and 1 mM, can stimulate the ERK1/2 phosphorylation; however, the p-GSK-3β level was up-regulated with 1 mM. Furthermore, the p-ERK1/2 up-regulation occurred in the lower dose (0.3 mM), and earlier than p-GSK-3β stimulation (Figure 4), which indicted the time-course activation of p-ERK1/2 signaling pathway. The p-ERK1/2 up-regulation was related to Li neuronal proliferation stimulation and protection against toxic agents (40). Moreover, Li pretreatment inhibits p-ERK1/2 expression and blocks AM antagonist on WIN induced changes in p-ERK1/2 expression. These results points toward a more complex effect for Li, since p-ERK1/2 was up-regulated by Li treatment alone, and was modulated with Li pretreatment against AM exposure. Li induces astrocyte G2/M cell cycle arrest and DNA synthesis with 1 mM concentration in a time- and dose-dependent manner, through inhibition of MEK-ERK phosphorylation with the activation of p38 MAP kinase in the stabilization of p53 induced by nitric oxide (NO) (40, 41), in an opposite effect on the neuronal induced proliferation (40), in consequence to the protective role of Li on brain injuries, and inhibited the cannabinoid withdrawal; and as a candidate it is potent pharmacotherapy for maintenance of bipolar and cannabis-use disorder patients. Study has demonstrated that the neuroprotective role of Li can occur through the activation of c-Jun N-terminal (JNK) and p38 kinase, as well as their downstream protein-1 (AP-1) DNA binding activation and p53 phosphorylation in glutamate neurotoxicity (42). Results showed that SL and Li pretreatment to WIN and AM can modulate the p-ERK1/2 up-regulation without down-regulation of the p-GSK-3β pathway in a time-dependent manner, since the highest inhibition occurred after 60 minutes of CGNs exposure. These data showed that ERK1/2 activation with AM was independent of GSK-3β in-activation to induce the cannabinoid-induced withdrawal. Li has affected Smad3/4-dependent transcriptional activity underlying the crosstalk of different signaling pathways through regulation of the cAMP/PKA and PI3-kinase/Akt/GSK-3β pathways (43).

Several biochemical alterations were observed during cannabinoid dependence, including up-regulation of AC/cAMP pathway (44). Cannabinoid-induced dependence/withdrawal was indicated by ELISA assay through the induction of cAMP production following FSK enhancement in study groups. Other signaling pathways related to NMDA receptor activation, which can be regulated with Cdk5, have mediated the development of cannabinoid physical dependence and the expression of cannabinoid withdrawal in animals, which can also be regulated with Li (45). NMDA receptor activation may enhance the cannabinoid dependence by increasing NO production in animals (46, 47). NO is one of the important intracellular second messengers that activates with NMDA receptor and oxidative stress status (48). NMDA receptor activation can increase the calcium influx to the neurons and the activation of nitric oxide synthase (NOS), which is responsible for NO production; and it was one of the molecular mechanisms which has been regulated with Li (49, 50). NO production has contributed to cannabinoid-induced withdrawal and can activate a number of second systems, including the guanylylcyclase (GC)-cyclic GMP system (PKG) (49). NO production has also been regulated with MEK/ERK1/2signaling pathways (51), which further suggest the involvement of ERK1/2 pathway in induction of cannabinoid-induced dependence. Therefore, cannabinoids can regulate two important second messengers of cGMP and cAMP, and they can be pioneered as cannabinoid abstinence indicators. However, more studies have persisted that cAMP production be utilized for this purpose (18).

AM, with a dose of 1 µM *in-vitro*, can not only antagonize the WIN cellular response, but also up-regulated the cAMP production, which may be an indicator of cannabinoid-induced dependence (17). It shows the antagonist/or inverse agonist effect of AM to WIN. CB1 receptors preferentially interact with G_αi_ to inhibit the AC/cAMP pathway (18). However, Li pretreatment can modulate the cAMP up-regulation against AM exposure. SL pretreatment can also inhibit the cAMP up-regulation against AM induction (Figure 8). It has also shown that cAMP production is induced by additional receptors other than CB1 or induces indirectly the modulation effect of Li against AM cellular response. These data have further suggested the potential role of Li pretreatment on the modulation of cannabinoid-induced dependence. Li alone can stimulate the cAMP/p-ERK1/2 pathway. These mechanisms are related to the protective role of Li against toxicants such as phencyclidine-induced neurotoxicity (52). However, when it was treated with AM and WIN, it modulated the cAMP production, which also indicated the protective role of Li on the changed of cAMP level, and protects neurons from being subjected to excitation/plasticity during the cannabinoid abstinence. Down-regulation of cAMP may arise due to a decrease in production or increased degradation through phosphodiestrase (PDEs). Li can compete with Mg^2+^, which is essential for AC activity, to lower cAMP formation in AC5-rich dopaminergic brain regions (53). Dopamine (DA) related systems, especially D_2_ receptors, are associated with drug abuse and addiction because their functions have been proven to be associated with drug reinforcement and relapses (54). Therefore, it can be involved in cannabinoid-induced dependence/withdrawal signs, which can be modulated by Li underlying cAMP regulation (55, 56). Since, Li can prevent the pathological changes in the expression of ERK1/2 signaling pathway through the modulation of cAMP cascade. Therefore, the dual effect of Li on the cAMP/p-ERK1/2 pathway can make the Li pretreatment a promising strategy for neuronal repair and to reduce cannabinoid withdrawal status; and it was suggested for the treatment of cannabis disorders. 

## Conclusion

Results confirmed that Li pretreatment with the dose of 1 mM could modulate the cAMP level as cannabinoid-induced dependence indicator against AM cellular response. The lower dose of 1 µM of WIN can up-regulate the p-GSK-3β and down-regulate the p-ERK1/2 and cAMP level. Therefore, cAMP, p-ERK1/2 and p-GSK-3β signaling pathways are important in development of cannabinoid-induced dependence. Furthermore, SL pretreatment can block the up-regulation of p-ERK1/2, without down-regulation of p-GSK-3β expression. Li pretreatment can also modulate the up-regulation of p-ERK1/2 and cAMP induced by AM exposure and up-regulation the p-GSK-3β, and therefore, inactivated of pro-apoptotic protein GSK-3β through stimulation of its phosphorylation. These data have demonstrated that Li pretreatment can modulate the cannabinoid-induced dependence/withdrawal signs possibly through regulations of cAMP/p-ERK1/2 cascade independent of p-GSK-3β signaling pathway in the CGNs-developed model. It is strongly showed the most neuroprotective effect of Li against cannabis-induced disorders. Therefore, evaluation of these pathways in animals will be the next step of our study. Furthermore, suitable clinical trial studies, which included Li to prevent cannabis-induced disorders or addiction, should be designed. However, not only ERK but also other PKs such as PI3K/Akt pathways may be relevant to cannabinoid receptor actions to induction of p-GSK-3β phosphorylation. Furthermore, more other receptors or molecular mechanisms may be involved in neuroprotective effect of Li to modulate the cAMP production against AM cellular response in which there are required to more evaluate in future studies.

## References

[1] Fakhfouri G, Ahmadiani A, Rahimian R, Grolla AA, Moradi F, Haeri A (2012). WIN55212-2 attenuates amyloid-beta-induced neuroinflammation in rats through activation of cannabinoid receptors and PPAR-gamma pathway. Neuropharmacol.

[2] Zogopoulos P, Vasileiou I, Patsouris E, Theocharis SE (2013). The role of endocannabinoids in pain modulation. Fundam. Clin. Pharmacol..

[3] Lafenetre P, Chaouloff F, Marsicano G (2007). The endocannabinoid system in the processing of anxiety and fear and how CB1 receptors may modulate fear extinction. Pharmacol. Res..

[4] Trazzi S, Steger M, Mitrugno VM, Bartesaghi R, Ciani E (2010). CB1 cannabinoid receptors increase neuronal precursor proliferation through AKT/glycogen synthase kinase-3beta/beta-catenin signaling. J. Biol. Chem..

[5] Fernandez-Ruiz J, Hernandez M, Ramos JA (2010). Cannabinoid-dopamine interaction in the pathophysiology and treatment of CNS disorders. CNS Neurosci. Ther..

[6] Orgado JM, Fernandez-Ruiz J, Romero J (2009). The endocannabinoid system in neuropathological states. Int. Rev. Psychiatry.

[7] Kuster JE, Stevenson JI, Ward SJ, D'Ambra TE, Haycock DA (1993). Aminoalkylindole binding in rat cerebellum: selective displacement by natural and synthetic cannabinoids. J. Pharmacol. Exp. Ther..

[8] Rezania F, Mehr SE, Kheirkhah M, Delfan B, Dehpour AR (2010). The effect of lithium chloride on WIN 55,212-2-induced tolerance in isolated guinea pig ileum. Eur. J. Pharmacol..

[9] Sim-Selley LJ (2003). Regulation of cannabinoid CB1 receptors in the central nervous system by chronic cannabinoids. Crit. Rev. Neurobiol..

[10] Castane A, Maldonado R, Valverde O (2004). Role of different brain structures in the behavioural expression of WIN 55,212-2 withdrawal in mice. Br. J. Pharmacol..

[11] Rahimian R, Dehpour AR, Fakhfouri G, Khorramizadeh MR, Ghia JE, Seyedabadi M, Caldarelli A, Mousavizadeh K, Forouzandeh M, Mehr SE (2011). Tropisetron upregulates cannabinoid CB1 receptors in cerebellar granule cells: possible involvement of calcineurin. Brain Res.

[12] Salavati P, Ramezani M, Monsef-Esfahani HR, Hajiagha R, Parsa M, Tavajohi S, Ostad SN (2013). Neuroprotective effect of total and sequential extract of Scrophularia striata Boiss. in rat cerebellar granule neurons following glutamate- induced neurotoxicity: an in-vitro study. Iran. J. Pharm. Res..

[13] Contestabile A (2002). Cerebellar granule cells as a model to study mechanisms of neuronal apoptosis or survival in-vivo and in-vitro. Cerebellum.

[14] Klein TW, Newton C, Larsen K, Lu L, Perkins I, Nong L, Friedman H (2003). The cannabinoid system and immune modulation. J. Leukoc. Biol..

[15] Howlett AC, Barth F, Bonner TI, Cabral G, Casellas P, Devane WA, Felder CC, Herkenham M, Mackie K, Martin BR, Mechoulam R, Pertwee RG (2002). International Union of Pharmacology. XXVII. Classification of cannabinoid receptors. Pharmacol. Rev..

[16] Sanchez MG, Ruiz-Llorente L, Sanchez AM, Diaz-Laviada I (2003). Activation of phosphoinositide 3-kinase/PKB pathway by CB(1) and CB(2) cannabinoid receptors expressed in prostate PC-3 cells. Involvement in Raf-1 stimulation and NGF induction. Cell Signal.

[17] Banafshe HR, Ghazi-Khansari M, Ejtemaei Mehr S, Dehpour AR (2007). Cyclosporine attenuates the adenylyl cyclase superactivation induced by chronic cannabinoid treatment. Eur. J. Pharmacol..

[18] Tzavara ET, Valjent E, Firmo C, Mas M, Beslot F, Defer N, Roques BP, Hanoune J, Maldonado R (2000). Cannabinoid withdrawal is dependent upon PKA activation in the cerebellum. Eur. J. Neurosci..

[19] Seely KA, Brents LK, Franks LN, Rajasekaran M, Zimmerman SM, Fantegrossi WE, Prather PL (2012). AM-251 and rimonabant act as direct antagonists at mu-opioid receptors: implications for opioid/cannabinoid interaction studies. Neuropharmacology.

[20] Rubino T, Forlani G, Vigano D, Zippel R, Parolaro D (2005). Ras/ERK signalling in cannabinoid tolerance: from behaviour to cellular aspects. J. Neurochem..

[21] Ellert-Miklaszewska A, Kaminska B, Konarska L (2005). Cannabinoids down-regulate PI3K/Akt and Erk signalling pathways and activate proapoptotic function of Bad protein. Cell Signal.

[22] Chiu CT, Chuang DM (2010). Molecular actions and therapeutic potential of lithium in preclinical and clinical studies of CNS disorders. Pharmacol. Ther..

[23] Chuang DM, Wang Z, Chiu CT (2011). GSK-3 as a target for lithium-induced neuroprotection against excitotoxicity in neuronal cultures and animal models of ischemic stroke. Front. Mol. Neurosci..

[24] Khaled M, Larribere L, Bille K, Aberdam E, Ortonne JP, Ballotti R, Bertolotto C (2002). Glycogen synthase kinase 3beta is activated by cAMP and plays an active role in the regulation of melanogenesis. J. Biol. Chem..

[25] Jope RS, Yuskaitis CJ, Beurel E (2007). Glycogen synthase kinase-3 (GSK3): inflammation, diseases, and therapeutics. Neurochem. Res..

[26] Huang HC, Klein PS (2006). Multiple roles for glycogen synthase kinase-3 as a drug target in Alzheimer's disease. Curr. Drug Targets.

[27] Seyedabadi M, Ostad SN, Albert PR, Dehpour AR, Rahimian R, Ghazi-Khansari M, Ghahremani MH (2012). Ser/ Thr residues at α3/β5 loop of Gαs are important in morphine-induced adenylyl cyclase sensitization but not mitogen-activated protein kinase phosphorylation. FEBS J.

[28] Valverde O, Maldonado R, Valjent E, Zimmer AM, Zimmer A (2000). Cannabinoid withdrawal syndrome is reduced in pre-proenkephalin knock-out mice. J. Neurosci..

[29] Vigano D, Rubino T, Parolaro D (2005). Molecular and cellular basis of cannabinoid and opioid interactions. Pharmacol. Biochem. Behav..

[30] Fratta W, Fattore L (2013). Molecular mechanisms of cannabinoid addiction. Curr. Opin. Neurobiol..

[31] Trazzi S, Steger M, Mitrugno VM, Bartesaghi R, Ciani E (2010). CB1 cannabinoid receptors increase neuronal precursor proliferation through AKT/glycogen synthase kinase-3beta/beta-catenin signaling. J. Biol. Chem..

[32] Ozaita A, Puighermanal E, Maldonado R (2007). Regulation of PI3K/Akt/GSK-3 pathway by cannabinoids in the brain. J. Neurochem..

[33] Stork PJ, Schmitt JM (2002). Crosstalk between cAMP and MAP kinase signaling in the regulation of cell proliferation. Trends Cell Biol.

[34] Cui SS, Bowen RC, Gu GB, Hannesson DK, Yu PH, Zhang X (2001). Prevention of cannabinoid withdrawal syndrome by lithium: involvement of oxytocinergic neuronal activation. J. Neurosci..

[35] Weinstein AM, Gorelick DA (2011). Pharmacological treatment of cannabis dependence. Curr. Pharm. Des..

[36] Daigle TL, Wetsel WC, Caron MG (2011). Opposite function of dopamine D1 and N-methyl-D-aspartate receptors in striatal cannabinoid-mediated signaling. Eur. J. Neurosci..

[37] Liao Y, Bin J, Luo T, Zhao H, Ledent C, Asakura M, Xu D, Takashima S, Kitakaze M (2012). CB1 cannabinoid receptor deficiency promotes cardiac remodeling induced by pressure overload in mice. Int. J. Cardiol..

[38] Diniz BS, Machado-Vieira R, Forlenza OV (2013). Lithium and neuroprotection: translational evidence and implications for the treatment of neuropsychiatric disorders. Neuropsychiatr. Dis. Treat..

[39] Wei YM, Li SX, Shi HS, Ding ZB, Luo YX, Xue YX, Lu L, Yu CX (2011). Protracted cocaine withdrawal produces circadian rhythmic alterations of phosphorylated GSK-3beta in reward-related brain areas in rats. Behav. Brain Res..

[40] Pardo R, Andreolotti AG, Ramos B, Picatoste F, Claro E (2003). Opposed effects of lithium on the MEK-ERK pathway in neural cells: inhibition in astrocytes and stimulation in neurons by GSK3 independent mechanisms. J. Neurochem..

[41] Nemeth ZH, Deitch EA, Szabo C, Fekete Z, Hauser CJ, Hasko G (2002). Lithium induces NF-kappa B activation and interleukin-8 production in human intestinal epithelial cells. J. Biol. Chem..

[42] Chen RW, Qin ZH, Ren M, Kanai H, Chalecka-Franaszek E, Leeds P, Chuang DM (2003). Regulation of c-Jun N-terminal kinase, p38 kinase and AP-1 DNA binding in cultured brain neurons: roles in glutamate excitotoxicity and lithium neuroprotection. J. Neurochem..

[43] Liang MH, Wendland JR, Chuang DM (2008). Lithium inhibits Smad3/4 transactivation via increased CREB activity induced by enhanced PKA and AKT signaling. Mol. Cell Neurosci..

[44] Rubino T, Vigano D, Massi P, Parolaro D (2000). Changes in the cannabinoid receptor binding, G protein coupling, and cyclic AMP cascade in the CNS of rats tolerant to and dependent on the synthetic cannabinoid compound CP55,940. J. Neurochem..

[45] Zhang S, Edelmann L, Liu J, Crandall JE, Morabito MA (2008). Cdk5 regulates the phosphorylation of tyrosine 1472 NR2B and the surface expression of NMDA receptors. J. Neurosci..

[46] Scott GS, Bowman SR, Smith T, Flower RJ, Bolton C (2007). Glutamate-stimulated peroxynitrite production in a brain-derived endothelial cell line is dependent on N-methyl-D-aspartate (NMDA) receptor activation. Biochem. Pharmacol..

[47] Rawls SM, Rodriguez T, Baron DA, Raffa RB (2006). A nitric oxide synthase inhibitor (L-NAME) attenuates abstinence-induced withdrawal from both cocaine and a cannabinoid agonist (WIN 55212-2) in Planaria. Brain Res.

[48] Rahimi HR, Shiri M, Razmi A (2012). Antidepressants can treat inflammatory bowel disease through regulation of the nuclear factor-kappaB/nitric oxide pathway and inhibition of cytokine production: A hypothesis. World J. Gastrointest. Pharmacol. Ther..

[49] Ghasemi M, Shafaroodi H, Nazarbeiki S, Meskar H, Ghasemi A, Bahremand A, Ziai P, Dehpour AR (2010). Inhibition of NMDA receptor/NO signaling blocked tolerance to the anticonvulsant effect of morphine on pentylenetetrazole-induced seizures in mice. Epilepsy Res.

[50] Ghasemi M, Dehpour AR (2011). The NMDA receptor/nitric oxide pathway: a target for the therapeutic and toxic effects of lithium. Trends Pharmacol. Sci..

[51] Cale JM, Bird IM (2006). Inhibition of MEK/ERK1/2 signalling alters endothelial nitric oxide synthase activity in an agonist-dependent manner. Biochem. J..

[52] Xia Y, Wang CZ, Liu J, Anastasio NC, Johnson KM (2008). Lithium protection of phencyclidine-induced neurotoxicity in developing brain: the role of phosphatidylinositol-3 kinase/Akt and mitogen-activated protein kinase kinase/ extracellular signal-regulated kinase signaling pathways. J. Pharmacol. Exp. Ther..

[53] Mann L, Heldman E, Bersudsky Y, Vatner SF, Ishikawa Y, Almog O, Belmaker RH, Agam G (2009). Inhibition of specific adenylyl cyclase isoforms by lithium and carbamazepine, but not valproate, may be related to their antidepressant effect. Bipolar Disord.

[54] Sharifzadeh M, Rezaei H, Ghamsari MR (2003). Interactive Effects of Acute and Chronic Lithium with Dopamine Receptor Antagonists on Naloxone-Induced Jumping in Morphine-Dependent Mice. Iran. J. Pharm. Res..

[55] Filip M, Zaniewska M, Frankowska M, Wydra K, Fuxe K (2012). The importance of the adenosine A(2A) receptor-dopamine D(2) receptor interaction in drug addiction. Curr. Med. Chem..

[56] Rahimi HR, Dehpour AR, Mehr SE, Sharifzadeh M, Ghahremani MH, Razmi A, Ostad SN (2014). Lithium attenuates cannabinoid-induced dependence in the animal model: involvement of phosphorylated ERK1/2 and GSK-3β signaling pathways. Acta Med. Iran..

